# Adverse Events in Healthy Individuals and MDR-TB Contacts Treated with Anti-Tuberculosis Drugs Potentially Effective for Preventing Development of MDR-TB: A Systematic Review

**DOI:** 10.1371/journal.pone.0053599

**Published:** 2013-01-11

**Authors:** Miranda W. Langendam, Edine W. Tiemersma, Marieke J. van der Werf, Andreas Sandgren

**Affiliations:** 1 Dutch Cochrane Centre, Academic Medical Center, University of Amsterdam, Amsterdam, The Netherlands; 2 KNCV Tuberculosis Foundation, The Hague, The Netherlands; 3 Center for Infection and Immunity Amsterdam (CINIMA), Academic Medical Center, University of Amsterdam, Amsterdam, The Netherlands; 4 European Centre for Disease Prevention and Control (ECDC), Stockholm, Sweden; McGill University, Canada

## Abstract

A recent systematic review concluded that there is insufficient evidence on the effectiveness to support or reject preventive therapy for treatment of contacts of patients with multidrug resistant tuberculosis (MDR-TB). Whether preventive therapy is favorable depends both on the effectiveness and the adverse events of the drugs used. We performed a systematic review to assess adverse events in healthy individuals and MDR-TB contacts treated with anti-tuberculosis drugs potentially effective for preventing development of MDR-TB. We searched MEDLINE, EMBASE, and other databases (August 2011). Record selection, data extraction, and study quality assessment were done in duplicate. The quality of evidence was assessed using the GRADE approach. Of 6,901 identified references, 20 studies were eligible. Among the 16 studies in healthy volunteers (a total of 87 persons on either levofloxacin, moxifloxacin, ofloxacin, or rifabutin, mostly for 1 week), serious adverse events and treatment discontinuation due to adverse events were rare (<1 and <5%, respectively), but mild adverse events frequently occurred. Due to small sample sizes of the levofloxacin and ofloxacin studies an increased frequency of mild adverse events compared to placebo could not be demonstrated or excluded. For moxifloxacin the comparative results were inconsistent. In four studies describing preventive therapy of MDR-TB contacts, therapy was stopped for 58–100% of the included persons because of the occurrence of adverse events ranging from mild adverse events such as nausea and dizziness to serious events requiring treatment. The quality of the evidence was very low. Although the number of publications and quality of evidence are low, the available evidence suggests that shortly after starting treatment the occurrence of serious adverse events is rare. Mild adverse events occur more frequently and may be of importance because these may provoke treatment interruption.

## Introduction

Multidrug-resistant tuberculosis (MDR-TB), defined as resistance to the two most effective anti-tuberculosis drugs isoniazid and rifampicin, is posing an enormous challenge to global TB control because of the long and complex treatment that is required to cure. The World Health Organization (WHO) estimated that in 2010, there were 650,000 cases of MDR-TB globally, accounting for 5.4% of all prevalent TB cases [1].

Like susceptible TB, MDR-TB can spread through expectoration and subsequent inhalation of infectious droplets. Spread of MDR-TB among household contacts of MDR-TB patients is well documented with a prevalence of MDR-TB in contacts ranging between 1.8% and 11.2% [2]–[11]. Also, MDR-TB outbreaks within health care facilities [12], schools [13], work places [14] and other confined settings [15] have been reported. Persons exposed to TB may develop latent TB infection and are at risk of developing TB disease. International guidelines recommend preventive therapy with isoniazid (INH) for 6 months for those with latent TB infection especially if co-infected with HIV [16],[17],[18]. However, this regimen cannot be applied to contacts of MDR-TB patients, since MDR-TB is resistant to INH by definition. Thus, alternative regimens are needed for the preventive treatment of MDR-TB.

The currently available guidelines on the management of contacts of MDR-TB patients provide conflicting recommendations. The WHO recommends to carefully follow-up close contacts of drug-resistant (DR-)TB patients for a period of at least two years without providing prophylactic treatment [19],[20]. The NICE guideline for clinical diagnosis and management of tuberculosis states that preventive treatment should not be given to contacts of MDR-TB patients [17]. In contrast, the Centers for Disease Control and Prevention of the United States of America do advise providing preventive chemotherapy with pyrazinamide in combination with ethambutol or a fluoroquinolone (depending on the resistance profile of the index-patient's strain) for 6–12 months to persons who are likely to be infected with MDR-TB and are at high risk of developing TB [16],[21],[22].

These conflicting recommendations can be explained by the lack of scientific evidence for the effectiveness of preventive therapy. In the most recent systematic review [23], three studies were identified that investigated the effectiveness of chemotherapy to prevent the development of MDR-TB [3],[6],[7]. The authors concluded that the studies provided insufficient evidence to support or reject preventive treatment for prevention of MDR-TB [23].

If preventive therapy would only be minimally effective (as indicated by one study among children in South Africa) [6] and safe (i.e. would not provoke any harmful adverse events), preventive chemotherapy could be considered as an option in the management of contacts of MDR-TB patients. Besides being harmful, (mild) adverse events that can lead to therapy discontinuation should occur rarely to ensure high treatment completion rates.

We performed a systematic review to summarize the evidence for the occurrence of adverse events to preventive treatment with anti-tuberculosis drugs other than isoniazid and rifampicin. To optimize applicability of the results, we only included clinical trials in healthy individuals and observational studies in contacts of MDR-TB patients.

This systematic review together with systematic review on effectiveness of preventive therapy in contacts of MDR-TB patients [23], are the evidence on which the recently launched European Centre for Disease Prevention and Control (ECDC) guidance on management of contacts of MDR-TB and XDR-TB patients is based [24].

## Materials and Methods

This systematic review followed the standards of the Cochrane Collaboration (Chapter 14 of the Cochrane Handbook) and PRISMA guidelines [25],[26].

### Search strategy

To identify relevant studies, we conducted a literature search in the bibliographic databases MEDLINE and EMBASE in August 2011. The following key words were used: names of individual TB drugs and synonyms for prevention and healthy individuals (e.g. contacts, latent, preventive). All antibiotics mentioned in the World Health Organization Guidelines for the programmatic management of drug-resistant tuberculosis [19],[20] were used, except for rifampicin and isoniazid and anti-tuberculosis drugs with unclear efficacy or unclear role in MDR-TB treatment (so-called ‘group 5-agents’) as these are not recommended by WHO for routine use in MDR-TB patients [19]. Furthermore, we applied an adverse events search filter which was based on the results of a study on developing efficient search strategies to identify reports of adverse events [27]. The search strategy is provided as Supporting Information S1.

The TRIP database (systematic reviews and guidelines) and BIOSIS Preview (conference abstracts) were searched in August 2011 using the keyword adverse event in combination with one of the drug names provided in the list above. The WHO International Clinical Trials Registry Platform (ongoing controlled trials) was also searched for each individual drug listed. We also searched for information on adverse events on drug label information via several websites: http://sideeffects.embl.de; http://www.tb.org.za (TB Online); http://www.tbonline.info; http://www.drugs.com.

To complete the search, hand searching the reference lists of the finally eligible studies was done.

The search protocol was developed by the authors in close collaboration with a clinical librarian.

### Eligibility criteria

Retrieved studies were either classified as experimental studies in healthy volunteers (randomized and non-randomized controlled trials) or as observational reports describing adverse events among contacts of MDR-TB patients and assessed for eligibility according to the criteria explained below.

We excluded studies in non-healthy individuals because adverse events might be more frequently accepted or ignored if given as treatment to sick patients, compared to what healthy individuals accept. The selected studies should as far as possible resemble the conditions of long term preventive therapy in healthy individuals (i.e. not suffer from TB or any other infectious disease for which the treatment is provided).

#### Experimental studies in healthy volunteers

We included randomized and non-randomized controlled trials reporting on adverse events to TB-drugs other than rifampicin, isoniazid and ‘group-5 agents’ (see ‘Search strategy’) in healthy individuals (healthy volunteers as according to the authors' description). Articles and reports on original data and published in English, German, Dutch, French, or Spanish were included.

Since preventive treatment is usually given for periods of at least several months, we were most interested in the longer term adverse events of treatment. Also, from studies identified in the recent systematic review on the effectiveness of preventive treatment [23] we had indications that most adverse events occur after the first week of treatment [12],[13],[28],[29]. Therefore, we excluded single dose studies and RCTs in which treatment was given for less than seven days. To be able to assess the adverse events per drug, we excluded studies in which combinations of drugs were administered and studies not giving details about the number and type of adverse events per drug. All types of adverse events were taken into account, except infusion site related adverse events for studies in which the drugs were administered intravenously since we assumed that preventive treatment would not be administered intravenously. “Serious” and “mild” adverse were defined as by the authors in the individual studies.

To judge whether the occurrence of adverse events is caused by the treatment, the best information available is from randomized placebo controlled studies. In addition, we also collected information from experimental studies that compared two or more different treatments (not placebo controlled) because this will give an indication about the absolute frequency of adverse events.

#### Studies in contacts of MDR-TB patients

Since our main interest was to assess the prevalence of adverse events among MDR-TB contacts receiving preventive MDR-TB treatment, we included observational studies reporting adverse events occurring among preventively treated contacts of MDR-TB patients. We included those studies describing a cohort of apparently healthy subjects and persons with co-morbidities that frequently occur in the general population (e.g. diabetes mellitus, myocardial infarction).

### Study selection

Eligible studies were selected in two phases. In the first phase all references in the database were reviewed for eligibility by two authors (MvdW and ML). References without an abstract were excluded, except when eligibility was clear from the title. In the second phase all potentially relevant articles were derived full-text and assessed for eligibility by applying the inclusion criteria (as stated in ‘Eligibility criteria’) by three authors (MvdW, ML and ET) independently. Inconsistencies were solved by discussion between the authors until consensus was reached.

### Data extraction and data analysis

Data were extracted using a data-extraction form (EpiData version 3.1, The EpiData Association, Odense, Denmark available at http://www.epidata.dk) independently by two authors (ML and ET). Inconsistencies were discussed to obtain consensus.

The risk of bias of included studies was assessed by two reviewers (ML and ET) independently using the Cochrane Risk of Bias tool for RCTs [25] and the Newcastle Ottawa Scale for the observational studies [30].

We planned to summarize the studies quantitatively; however, the studies were too heterogeneous and were therefore summarized qualitatively. If the study included more than one study arm with the drug of interest, for example different doses or different modes of administration, the results are presented for each study arm. The results are presented as frequency of the occurrence of adverse events. For comparisons between intervention and placebo a relative risk and 95% confidence intervals are presented.

The quality of the evidence was assessed using the GRADE approach [31]. We assessed the quality of evidence for the overall outcome ‘adverse events’. The GRADE approach considers the results from RCTs as high quality evidence. However, study limitations, indirectness, inconsistency, imprecision or publication bias makes downgrading to a lower quality level (moderate, low or very low) necessary. The results of observational studies are considered as low quality evidence, however there are criteria for rating up the quality level: a large effect (at least a two-fold increase or reduction in risk), a dose-response gradient and if all plausible confounding would decrease an apparent treatment effect, or in case of no effect, would create a spurious effect. The quality of evidence was assessed by intervention (comparison) and outcome, across studies.

## Results

### Search strategy and selection process

**Figure 1:**
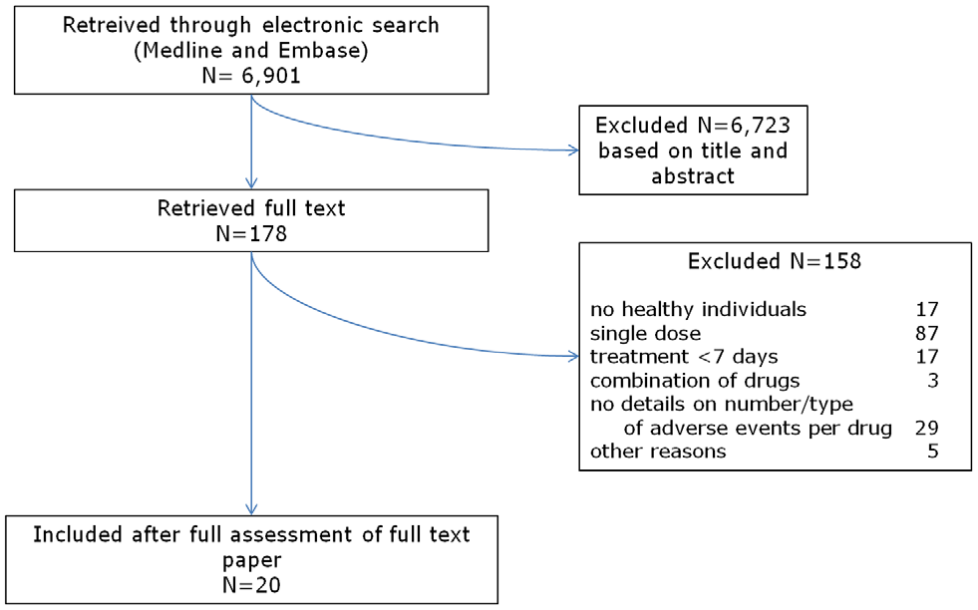
Systematic review process flowchart.

In total we retrieved 6,901 records from MEDLINE and EMBASE. After duplicate assessment of title and abstract of these records, 178 references were left for full-text assessment. Applying the inclusion criteria, we excluded another 158 papers. All excluded reports described experimental studies. The reasons for exclusion were: no healthy individuals (n = 17), single dose study (n = 87), less than seven days treatment (n = 17), combination of drugs administered in all arms (n = 3), no details about number and type of adverse events per drug (n = 29), language (n = 1), no original data (n = 3) and duplicate publication (n = 1). Twenty papers were included (PRISMA flowchart Figure 1).

There were no additional results from searching the TRIP database (35 systematic reviews and guidelines), BIOSIS Preview (90 conference abstracts were found) and reference tracking. The search using the WHO International Clinical Trials Registry Platform (ongoing controlled trials) resulted in 30 ongoing studies. These ongoing controlled trials are mainly moxifloxacin studies. In the search for data on adverse events on drug label information we found information for kanamycin, amikacin, capreomycin, streptomycin, ethionamide, terizidone and p-aminosalicylic acid (PAS). The label information did not include relevant literature references.

### Included studies: characteristics

Of the 20 selected studies, 16 were experimental studies in healthy volunteers and 4 were observational studies in contacts of MDR-TB patients.

#### Experimental studies in healthy volunteers

Fourteen RCTs [32]–[45] and two single arm studies [46],[47] were included (Table 1).

**Table 1:** Characteristics of included studies.^a^

First author, Year (reference)	Funding	Study type	Comparison	Methods of AE assessment	Dose (mg); IV or oral	Sample size Treatment; comparison	Treatment duration (washout period)
*Levofloxacin*							
Amsden, 1999 [32]	Industry	cross-over RCT	alatrofloxacin 200 mg	non-systematic active+passive	500; IV	12	7 (14)
Chien, 1998 [33]	NR	parallel RCT	placebo	non-systematic active+passive	750; oral	10 ; 6	7
Chien, 1998 [33]	NR	parallel RCT	placebo	non-systematic active+passive	1000; oral	10 ; 6	13
Chien, 1997 [34]	Industry	parallel RCT	placebo	non-systematic active+passive	500; oral	10 ; 10	7
Chien, 1997 [34]	Industry	parallel RCT	placebo	non-systematic active+passive	500; IV	10 ; 10	7
Chow, 2001 [35]	NR	parallel RCT	placebo	systematic objective+non-systematic active	750; IV	12 ; 6	7
Tsikouris, 2006 [36]	NR	cross-over RCT	ciprofloxacin 1000 mg	not defined	400; oral	13	7 (7)
Zhang, 2002 [47]	NR	single arm	no comparison	not defined	200; IV	10	7
*Moxifloxacin*							
Ayalasomayajula, 2008 [37]	Industry	parallel RCT	placebo	systematic objective+non-systematic active+passive	400; oral	76 ; 77	7
Burkhardt, 2002 [38]	NR	cross-over RCT	clarithromycin 500 mg	systematic objective+non-systematic active	400; oral	12	7 (42)
Peeters, 2008 [39]	Industry	cross-over RCT	placebo	systematic objective+non-systematic active	400; oral	41	8
Sullivan, 1999 [40]	NR	parallel RCT	placebo	systematic subjective+systematic objective+non-systematic active+passive	400; oral	10 ; 5	10
Tsikouris, 2006 [36]	NR	cross-over RCT	ciprofloxacin 1000 mg	not defined	400; oral	13	7
*Ofloxacin*							
Guay, 1992 [41]	Industry	parallel RCT	placebo	systematic objective	200; IV	12 ; 12	7
Guay, 1992 [41]	Industry	parallel RCT	placebo	systematic objective	400; IV	12 ; 12	7
Marier, 2006 [42]	Industry	cross-over RCT	ofloxacin 200 mg	non-systematic active	400; oral	40	7 (7)
Marier, 2006 [42]	Industry	cross-over RCT	ofloxacin 400 mg	non-systematic active	200; oral	40	7 (7)
Stein, 1991 [43]	NR	parallel RCT	placebo	systematic subjective+systematic objective+non-systematic active+passive	400; oral	12 ; 12	10
Van Saene, 1988 [46]	NR	single arm	no comparison^b^	not defined	200; oral	15	7
*Rifabutin*							
Ford, 2008 [44]	Industry	cross-over RCT	fosamprenavir 700 mg+ritanovir 100 mg	systematic objective+non-systematic active	300; oral	17	13 (21)
Kraft, 2004 [45]	Industry	cross-over RCT	rifabutin placebo and indinavir	not defined	300; oral	13	10 (10)
Kraft, 2004 [45]	Industry	cross-over RCT	indinavir 800 mg	not defined	300; oral	20	10 (10)
*Pyrazinamide+Ethambutol*							
Younossian, 2005 [29]	NR	case series of definite contacts of MDR-TB patients with positive TST	no comparison	not defined	PZA 23±4 mg/kg+EMB 17±4 mg/kg; oral	12	87^c^
*Pyrazinamide+Levofloxacin*							
Papastavros, 2002 [28]	NR	case series of definite contacts of MDR-TB patients with positive TST	no comparison	systematic subjective+systematic objective	PZA 15–17 mg/kg+LVX 500–750 mg/kg; NR	17	20–38; median 32
*Pyrazinamide+Ofloxacin*							
Horn, 1994 [12]	NR	case series of HCW caring for MDR-TB patients with documented recent TST conversion	no comparison	not defined	PZA 1500+OFX 800; oral	16	90^d^
Ridzon, 1997 [13]	government	case series of contacts of MDR-TB patients with positive TST (21 of 22 with documented recent TST conversion)	no comparison	systematic objective+systematic subjective	PZA 25 mg/kg+1× OFX 600 mg; oral	22	365^e^

a NR = not reported; RCT = randomized controlled trial; IV = intravenous; USA = United States of America; TST = tuberculosis skin test (Mantoux test); EMB = ethambutol; PZA = pyrazinamide; LVX = levofloxacin; HCW = health care workers; OFX = ofloxacin.

b 6 of 15 volunteers were treated with ciprofloxacin 2 months after having been treated with ofloxacin. Because the order of treatments was fixed and only 40% of the participants agreed to participate in the second phase of the study, we did not consider the ciprofloxacin treatment period as a comparison for this report.

c Minimum duration of treatment, intended duration of treatment was 9 months.

d Median duration of treatment, intended duration was 6 months.

e Medications were stopped prematurely for 13 out of the 17 participants.

Six studies on levofloxacin were included [32]–[36], [47] (eight study arms; three RCTs were placebo controlled [33]–[35]), five RCTs studied moxifloxacin [36]–[40] (five study arms; three placebo controlled [37],[39],[40] though the first of these three was blinded for the drug of primary interest, aliskiren, but not for moxifloxacin [37]), four reports were on ofloxacin [41]–[43], [46] (six study arms; two placebo controlled [41],[43]), two RCTs studied rifabutin [44],[45] (three study arms; none placebo controlled).

Seven out of 16 studies included more than 80% male volunteers. No studies included children or adolescents (age below 18 years). All but one study had small sample sizes, ranging between 10 and 41 participants. One RCT randomized 298 persons, of which 76 received moxifloxacin [37]. In 15 of 22 study arms (68%) the volunteers were treated for seven days [32]–[38],[41],[42],[46],[47] and in seven (32%) for 8 to 13 days [33],[39],[40],[44],[45].

#### Studies in contacts of MDR-TB patients

In the studies on preventive MDR-TB treatment combined drug treatment was used: pyrazinamide and ethambutol (one study, [29]), pyrazinamide and levofloxacin (one study, [28]) and pyrazinamide and ofloxacin (two studies, [12],[13]).

Three studies included both men and women in approximately equal proportions [13],[28],[29]. The median age of the participants was around 35 in two reports [28],[29] but only 17 in one [13]. One report does not provide details about sex and age of the cohort [12].

Due to their nature, all these studies had small sample sizes, ranging from 12 [29] to 22 [13] individuals.

The intended treatment duration of the studies in latent TB cases varied between 6 and 12 months. Due to high dropout rates however, the median duration of treatment was one month in one study [28] and three months in two other studies [12],[29]. One report did not report the median duration of treatment [13].

### Assessment of risk of bias in individual studies

A detailed description of the Risk of Bias assessment can be found as Supporting Information S2.

#### Experimental studies in healthy volunteers

Only two of the 14 included RCTs reported having applied random sequence generation for randomly assigning treatments [39],[42] whereas allocation concealment was not described in any of the other trials. Six trials were at high risk of bias because of lack of blinding of study staff and/or participants [32],[36],[37],[42],[44],[45]. These were all actively controlled trials. Beforehand knowledge about the potential adverse events may have caused bias in reporting adverse events, although the direction of the bias (under- or over reporting) remains unclear.

The single arm studies in healthy volunteers had some methodological problems. Van Saene and colleagues did not describe how they assessed that the volunteers were healthy, and the target population from which the volunteers were selected was not described [46]. Furthermore, they did not describe how outcome was assessed, which was the same for Zhang *et al* [47].

#### Studies in contacts of MDR-TB patients

The studies in contacts of MDR-TB patients were of high methodological quality. Although one report only included high school teachers and students [13] and another report described an outbreak including health care workers only [12], we judged all study populations to be at least somewhat representative of an average contact of an MDR-TB patients, since outbreaks involving large numbers of contacts to be treated preventively occur in such settings relatively frequently. All subjects had been exposed, confirmed by an epidemiological link in combination with TST positivity [28],[29] or seroconversion TST conversion [12],[13]. All reports stated that the outcome of interest was not present before the start of the study. There was a detailed description of outcome assessment in all reports but one [12], and all subjects were followed up for an adequate length of time, that is, at least 3 months.

### Effects of the interventions

The adverse events of the various treatment regimens are summarized in tables 2 and 3.

**Table 2:** Adverse events reported in the studies in healthy volunteers.

LEVOFLOXACIN										
	Serious^a^			Reason for dropout			Mild			
	Treat-ment n (%)	Placebo.n (%)	RR (95% CI)	Treat-ment n (%)	Placebo.n (%)	RR (95% CI)	Treat-ment n (%)	Placebo.n (%)	RR (95% CI)	Type
***Placebo controlled***										
Chien, 1998 [33].750 mg; oral	NR	NR	-	0/10	1/6^b^	0.21 (0.01–4.51)	3/10 (30.0)	3/6 (50.0)	0.60 (0.17–2.07)	Headache and nausea in treatment and placebo group
Chien, 1998 [33] 1 g; oral	NR	NR	-	0/10	1/6^b^	0.21 (0.01–4.51)	4/10 (40.0)	3/6 (50.0)	0.80 (0.27–2.41)	Headache and nausea in treatment and placebo group
Chien, 1997 [34].500 mg; oral	0/10	0/10	-	0/10	0/10	-	2/10 (20.0)	0/10	5.00 (0.27–92.62)	Treatment: abdominal pain and dizziness
Chien, 1997 [34] 500 mg; IV	0/10	0/10	-	0/10	0/10	-	0/10	0/10	-	-
Chow, 2001 [35].750 mg; IV	NR	NR	-	0/12	0/6	-	4/12 (25.0)	3/6 (50.0)	0.67 (0.22–2.07)	Treatment: dizziness, head-ache, euphoria. Placebo: headache, dizziness, purpura
***Not placebo controlled***										
Amsden, 1999 [32] 500 mg; IV	0/12			0/12			4/12 (33.3)			Dizziness, feeling of faintness, headache, lightheadedness
Tsikouris, 2006 [36] c 400 mg; oral	0/13			0/13			0/13 (0.0)			-
Zhang, 2002 [47] c 200 mg; IV	0/10			0/10			1/10 (10.0)			Transitory phlebitis (probably related to infusion)

MOXIFLOXACIN										
	Serious^a^			Reason for dropout			Mild			
	Treat-ment n (%)	Placebo n (%)	RR (95% CI)	Treat-ment n (%)	Placebo n (%)	RR (95% CI)	Treat-ment n (%)	Placebo n (%)	RR (95% CI)	Type
***Placebo controlled***										
Ayalasomayajula, 2008 [37] 400 mg; oral	0/76	0/77	-	1/76 (1.3)^d^	2/77 (2.6)^e^	0.51 (0.05–5.47)	26/76 (34.2) 20/76 (26.3)	13/77 (16.9) 16/77 (20.8)^f^	2.03 (1.13–3.64) 1.27 (0.71–2.25)	Gastrointestinal events (diarrhea, nausea, loose stools) Nervous system disorders (headache, dizziness)
Peeters, 2008 [39] 400 mg; oral	1/39^i^	0/39	3.00 (0.13–71.46)	0/39	0/39	-	23/39 (59.0)	22/39 (56.4)	1.05 (0.71–1.53)	Most common AE Treatment (17 events): headache, erythema, pharyngolaryngeal pain, abdominal pain, rash, cardiac events. Placebo (28 events): headache, erythema, pruritus, abdominal pain, rash, cardiac events
Sullivan, 1999 [40] 400 mg; oral	NR	NR	-	0/10	0/5	-	8/10 (80.0)	4/5 (80.0)	1.00 (0.58–1.71)	In both groups: abdominal pain, headache, diarrhea, nausea, dizziness. Treatment: arm pain, dry mouth, dyspepsia, flatulence, tooth pain, rhinitis, acne
***Not placebo controlled***										
Burkhardt, 2002 [38] 400 mg; oral	0/12			0/12			6/12 (50.0) 3/12 (25.0) 4/12 (33.3) 3/12 (25.0)			Flatulence Taste perversion Diarrhea Headache
Tsikouris, 2006 [36] 400 mg; oral	0/13			0/13			7/13 (53.8) 2/13 (15.4) 1/13 (7.7)			Nausea Lightheadedness Dry mouth

OFLOXACIN										
	Serious^a^			Reason for dropout			Mild			
	Treat-ment n (%)	Placebo n (%)	RR (95% CI)	Treat-ment n (%)	Placebo n (%)	RR (95% CI)	Treat-ment n (%)	Placebo n (%)	RR (95% CI)	Type
***Placebo controlled***										
Guay, 1992 [41] 200 mg; IV	NR	NR	-	2/12 (16.7)^g^	0/12	5.00 (0.27–94.34)	8/12 (67.7)	8/12 (67.7)	1.00 (0.57–1.76)	Treatment (49 events): extremity pain, dizziness, headache, paresthesia, ocular dryness/redness, edema, tachycardia, abnormal chest sounds, diarrhea, urinary incontinence, skin erythema, rash. Placebo (32 events): chills, trunk pain, extremity pain, headache, syncope, edema, gastrointestinal cramps/distress, diarrhea, skin lesions, skin erythema
Guay, 1992 [41] 400 mg; IV	NR	NR	-	0/12	0/12	-	5/12 (41.7)	3/12 (25.0)	1.67 (0.51–5.46)	Treatment (16 events): dizziness, headache, otalgia, vasodilatation, pharyngitis, constipation, keratoderma, rash. Placebo (8 events): dizziness, tremor, dream abnormalities, dyspnea, pharyngitis, rash, arthralgia
Stein, 1991 [43] 200 mg; oral	0/12	0/12	-	0/12	0/12	-	8/12 (67.7)	7/12 (58.3)	1.14 (0.61–2.13)	Treatment (25 events): headache, visual disturbance, respiratory congestion, GI upset, nausea, vomiting, constipation, diarrhea, dry mouth.Placebo (15 events): headache, pyrexia, respiratory congestion, rhinitis, pharyngitis, GI upset, dry mouth
***Not placebo controlled***										
Marier, 2006 [42] 200 mg; oral	0/40	0/40	-	2/40^h^			28/40 (70.0)			Abdominal pain/cramps, nausea, stomach pain, loss of appetite, vomiting, constipation, bloated feeling, headache, dizziness, feeling weakness, hiccups, loose stools
Marier, 2006 [42] 400 mg; oral	0/40	0/40	-	0/40			30/40 (75.0)			Abdominal pain/cramps, stomach pain, loss of appetite, vomiting, flatulence, headache, dizziness, feels hot, burning in throat, loose stools, nausea
Van Saene, 1988 [46] c 200 mg; oral	NR	NR		0/15			0/15			

RIFABUTIN										
	Serious^a^			Reason for dropout			Mild			
	Treat-ment n (%)	Placebo n (%)	RR (95% CI)	Treat-ment n (%)	Placebo n (%)	RR (95% CI)	Treat-ment n (%)	Placebo n (%)	RR (95% CI)	Type
***Not placebo controlled***										
Ford, 2008 [44] 300 mg; oral	0/17			4/17 (23.5)^i^			12/17 (70.6)			Headache, chromaturia, diarrhea, neutrophil count decreased, pain, neutropenia, insomnia
Kraft, 2004 [45] (RIF+placebo) 300 mg; oral	0/13			1/13 (7.7)^j^			4/13 (30.8)			Most common: taste perversion
Kraft, 2004 [45] (RIF alone) 300 mg; oral	0/20			0/20			1/20 (5.0)			Vaginitis

Treat = treatment; NR = not reported; RR = relative risk; CI = confidence interval; IV = intravenous; AE = adverse effects.

a as reported by authors.

b reason dropout not reported.

c very limited reporting adverse events.

d backache.

e dizziness; lightheadedness.

f overall percentage (all groups).

g rash; dizziness and tachycardia.

h moderate headache with increasing intensity; repeated vomiting.

i moderate rash (n = 3); mild nausea.

j liver function and hematologic abnormalities.

**Table 3:** Adverse events (AE) reported in the studies in contacts of MDR-TB patients.

Treatment	Number of serious AE^a^	Number of AE that were reason for dropout	Adverse events
**PYRAZINAMIDE+ETHAMBUTOL**			
Younossian, 2005 [29]	0/12	7/12 (58%)	7/12 (58%) discontinued because of increase in ASAT or ALAT (n = 6) or mild elevation of liver enzymes associated with gastrointestinal symptoms. Symptoms: nausea (2/12); vomiting (1/12); loss of appetite (1/12); dizziness (1/12); visual disturbances with normal VEP (1/12); increased ALAT or ASAT (5/12).
**PYRAZINAMIDE+LEVOFLOXACIN**			
Papastavros, 2002 [28]	0/12	17/17 (100%)	17/17 (100%) experienced at least one abnormal symptom or sign and both drugs were discontinued in all patients. Profiles: gastrointestinal disorders (9/17); nervous system disorders (8/17); hyperuricemia (uric acid+urate level>upper limit of normal) (8/17); elevated liver enzymes (8/17); dermatological (5/17); musculoskeletal disorders (14/17).
**PYRAZINAMIDE+OFLOXACIN**			
Horn, 1994 [12]	NR	14/16 (88%)	13/17 had one or more adverse effects: gastrointestinal distress (6/16); insomnia (3/16); vertigo (2/16); arthralgia (7/16); hepatitis requiring treatment (4/16); pruritus (4/16); fatigue (4/16); rash (3/16); increased ALAT levels (4/16). Previous use of INH may have contributed to development of hepatitis.
Ridzon, 1997 [13]	3/22	13/22 (59%)	Medications were stopped for 13 contacts: 7/13 had mild to moderate increases in serum aminotransferase levels. Adverse events: nausea (3/22); diarrhea (1/22); persistent vomiting (1/22); lost appetite (1/22); angioedema (1/22*); arthralgia (2/22); itching (2/22); fatigue (1/22); sour taste in mouth (1/22); feeling hot and tingling (1/22); elevated ASAT/ALAT (mild: 9/22, significant: 2/22*)). * serious adverse events

a as reported by authors.

For the placebo-controlled studies, we calculated the relative risk (and 95% confidence interval) for the difference in the frequency of adverse events. Because of considerable clinical heterogeneity (measurement of adverse events) the results could not be pooled.

In general, the number of serious adverse events and adverse events that were reason for dropout were very low.

#### Experimental studies in healthy volunteers (table 2)

*Levofloxacin:* None of the included studies [32]–[36],[47] reported any serious adverse events, adverse events that needed treatment or dropout related to adverse events. The most commonly reported adverse events were dizziness, headache, nausea and abdominal pain. These events occurred with both administration routes and at different doses given.

Compared to placebo (3 studies, 5 comparisons), levofloxacin does not seem to evoke a higher percentage mild adverse events, but due to low sample sizes the confidence intervals were very wide. The frequency of occurrence of the mild adverse effects seems to increase with dosage.

*Moxifloxacin:* One serious adverse event that needed treatment was reported (in 39 participants) though this was considered as probably unrelated to the treatment [39]. In another study one out of 76 participants discontinued treatment because of adverse events [37].

Reported mild adverse events of moxifloxacin were primarily gastrointestinal (diarrhea, nausea, flatulence and abdominal pain), headache and dizziness.

The comparative results were inconsistent. In one of the three placebo-controlled studies the data suggest that mild gastrointestinal adverse events occur twice as often after use of moxifloxacin compared to placebo (RR 2.03, 95% CI 1.13–3.64) [37]. In the other two studies however, no such effect was found [39],[40]. In these two studies the percentage adverse events was higher compared to the first study [37].

*Ofloxacin:* Marier and colleagues reported that two of 40 participants were withdrawn from the study because they needed treatment of an adverse event; one of these had completed the 7-day 200 mg daily regimen and was on the second day of the 400 mg daily regimen when withdrawn because of repeated vomiting, whereas the other one needed treatment for dizziness and headache of increasing severity halfway the second period of treatment (order of regimens not stated) [42]. Adverse events leading to discontinuation of intravenous administration of 200 mg ofloxacin per day occurred in two out of 12 participants because of rash, respectively dizziness and tachycardia [41].

Frequently reported mild adverse events from these studies were gastrointestinal events, headache and dizziness. Gastrointestinal events were diarrhea, constipation, abdominal pain and nausea; the latter two being reported by participants receiving oral doses of ofloxacin [42],[43]. One study did not report any adverse events [46]. The percentage mild adverse events in the ofloxacin group varied between 42 and 75% for the different studies.

The sample sizes of the placebo-controlled studies [41],[43] were very low. For both studies the confidence intervals were very wide; there were no statistically significant differences between the groups.

*Rifabutin:* Two participants (1/13 and 1/20 from one study with two study arms [45]) discontinued treatment because of adverse events: one of these had an unspecified laboratory adverse event, but the treatment given at withdrawal was not reported, while the other person had a decrease in circulating neutrophils during washout after completion of the rifabutin treatment.

In one study 71% of the participants experienced adverse events [44], while in the other study the percentage adverse of events was much lower.

There were no placebo-controlled studies.

#### Studies in contacts of MDR-TB patients (table 3)

Four studies reported on the preventive treatment of (possible) latent tuberculosis infection of MDR-TB contacts. Combination therapy was prescribed for 6 to 12 months. All used pyrazinamide with another drug (ofloxacin in two studies [6],[7], ethambutol in one [10] and levofloxacin in another study [9]).

All these studies reported a high frequency of adverse events. Treatment was discontinued in 58–100% of the subjects due to adverse events ranging from mild adverse events such as nausea and dizziness to serious events requiring treatment.

### Quality of the evidence

The GRADE profiles for levofloxacin, moxifloxacin, ofloxacin and rifabutin are presented in detail as Supporting Information S3. In summary, we downgraded the experimental studies one level for study limitations (lack of blinding, limited duration of treatment, no comparison), one or two levels for indirectness (healthy volunteers instead of contacts of MDR-TB patients, different dosages, intravenous instead of oral treatment) and one level for imprecision (small sample size and low number of events). For each drug this results in the rating ‘very low quality evidence’.

The observational studies provide a direct answer, but because of the non-comparative design the quality of the evidence is very low. Therefore the overall quality of the evidence for occurrence of adverse events related to chemoprophylaxis in contacts of MDR-TB patients is very low.

## Discussion

### Summary of main results

This systematic review assessed the occurrence of adverse events of anti-TB drugs that can be used for the prevention of TB disease in contacts of MDR-TB patients. We found 16 studies in healthy volunteers receiving levofloxacin, moxifloxacin, ofloxacin and rifabutin and 4 studies on pyrazinamide in combination with ethambutol, levofloxacin or ofloxacin in contacts of MDR-TB patients. The studies in healthy volunteers [32]–[47] showed that serious adverse events were not frequently reported when drugs were given for a mean of 7 days. Only one study, on moxifloxacin, reported one event in 39 participants (probably unrelated to the treatment). Mild adverse events occurred relatively frequent, in up to 70 to 80 percent of the healthy volunteers, however the frequencies varied highly between the studies.

Due to small sample sizes of the levofloxacin and ofloxacin studies an increased frequency of mild adverse events compared to placebo could not be demonstrated or excluded. For moxifloxacin the comparative results were inconsistent.

In the studies in contacts of MDR-TB patients treatment was scheduled for six months, but was discontinued in 58–100% of the subjects due to adverse events, which ranged from mild adverse events such as nausea and dizziness to serious events requiring treatment.

Using the GRADE method, we assessed the quality of the evidence as very low.

### Overall completeness and applicability of evidence

Although our search strategy was extensive and included all anti-TB drugs on the WHO list we did not identify any studies on capreomycin, para-aminosalycilic acid (PAS), protionamide, streptomycin, and terizidone. According to the WHO International Clinical Trials Registry Platform, RCTs in healthy volunteers are currently being undertaken on terizidone, moxifloxacin, levofloxacin, rifabutin, D-cycloserine, and ethambutol; so more information on adverse events caused by these drugs may become available soon. We did identify studies on amikacin, cycloserin, ethambutol, ethionamide, gatifloxacin, kanamycin and pyrazinamide treatment, but these studies were single dose studies or studies performed in non-healthy individuals.

Publication bias may also lead to incomplete evidence, since studies on drugs with no or limited effects on the outcome of interest and many adverse events will be less likely to be published than studies showing an important main effect. On the other hand, published cohort studies or case series addressing adverse events may give an overestimation. Preventive treatment of contacts of MDR-TB patients has been described in the published literature of MDR-TB outbreaks without report of adverse events (e.g., [15],[48]). Probably, only if treatment provokes many adverse events and a high prevalence of therapy discontinuation, special attention will be given to the treatment's adverse events.

Drug label information might be another source to complete the evidence on potential adverse events. However, drug labels often do not give information about the source and frequency of adverse events and therefore this information is not included in this systematic review.

The quality of the data, i.e. the reporting of adverse events, including how adverse events were defined and measured, was often inadequate to make a valid analysis. For example, some of the included studies only reported the most frequently occurring adverse events.

The sample size of all included studies in this review is too small to identify very uncommon, very serious adverse events. It is important to capture rare adverse events as well, because preventive treatment is only warranted if there is no significant risk to the patient of serious harm or death.

In summary, the reasons that the current numbers are low are most likely that (a) anti-TB drugs are understudied; and (b) “healthy volunteers” generally means Phase I trials, and therefore small numbers.

From the viewpoint of applicability, we decided not to include studies in which the drugs were administered to patients with a certain illness. For this reason, data on pharmacovigilance were not informative. With regard to applicability of the included evidence, most of the included studies provided treatment for 7 days [32]–[38],[41],[42],[46],[47] whereas anti-TB prophylaxis involves treatment for 3 months to 1 year. The observational studies for which the authors provided details about the onset of adverse events [13],[28],[29] generally showed that adverse events usually started after a few weeks of treatment. For example, Younossian and co-authors showed that about 90% of the 37 adverse events occurred after more than one week of treatment. For this reason, and because of the difference in study population, the results of studies providing only one week of treatment cannot be easily extrapolated to long-term treatment regimens.

Most studies were in a relatively homogeneous group of individuals: healthy volunteers with limited age range, exclusion of pregnant women, children and elderly. Real contacts will include all age groups and people with diverse comorbidity.

If a preventive chemotherapeutic regimen is considered, it should be noted that also mild adverse events such as diarrhea, headache and rash or itching may lead to discontinuation of therapy. Studies in which preventive chemotherapy was offered to contacts of MDR-TB patients invariably show that mild adverse events, occurring alone or in combination with other mild adverse events, can lead to interruption of treatment, irrespective of the type of drugs given. Also, these studies show that adverse events are very common and result in the discontinuation treatment for the majority of the participants. Note that all regimens included pyrazinamide which is known as hepatotoxic since its early days [49].

To provide more good quality data on adverse events due to preventive anti-TB treatment RCTs in individuals with LTBI are needed. Information on adverse events and interruption of preventive treatment that is already given for MDR-TB should be collected centrally using structured and protocolized formats.

## Conclusions

Clinicians who consider provision of preventive treatment to a contact of an MDR-TB case need to perform a comprehensive risk assessment of the contact. This assessment should take into account the individual risk for developing TB disease, the drug resistance pattern of the presumed source case, and the risk for adverse events caused by preventive treatment. However, the evidence on the occurrence of adverse events of anti-TB drugs in healthy persons is limited and of low quality and is too scarce to support or reject preventive therapy in MDR-TB and XDR-TB contacts. The alternative to giving preventive therapy is to provide counseling and follow-up by close observation of MDR-TB contacts. Overall, serious adverse events are infrequent.

## Supporting Information

Supporting Information S1 **Search strategy.**
(DOCX)

Supporting Information S2 **Risk of bias assessment.**
(DOC)

Supporting Information S3 **GRADE profiles.**
(DOCX)

Supporting Information S4 **PRISMA checklist.**
(DOC)
