# Supplementary material for: Adverse Events in Healthy Individuals and MDR-TB Contacts Treated with Anti-Tuberculosis Drugs Potentially Effective for Preventing Development of MDR-TB: A Systematic Review
Source: PLoS One. 2013 Jan 11;8(1):e53599. doi: 10.1371/journal.pone.0053599 (PMC3543458; doi:10.1371/journal.pone.0053599)
Supplement: Supporting Information S1 — Search strategy. (DOCX) [file pone.0053599.s001.docx]

Search strategy

A. Medline

Platform: OvidSP

Database: Ovid MEDLINE(R) In-Process & Other Non-Indexed Citations and Ovid MEDLINE(R) 1948 to Present

Date: 23-08-2011

Limits: no limits were used

Methodological filters: Adverse effect filter: Golder, S. et al Developing efficient search strategies to identify reports of adverse effects in Medline and Embase. Health information and libraries journal (2006),23, p3-12

| **Line #** | **Terms** |
| --- | --- |
| 1. | latent.mp. |
| 2. | contact*.mp. |
| 3. | exp Contact Tracing/ |
| 4. | exp Antibiotic Prophylaxis/ |
| 5. | exp Chemoprevention/ |
| 6. | prevent*.mp. |
| 7. | prophyla*.mp. |
| 8. | or/1-7 |
| 9. | exp Ethambutol/ |
| 10. | ethambutol.ti,ab. |
| 11. | pyrazinamide.mp. or exp Pyrazinamide/ |
| 12. | streptomycin.mp. or exp Streptomycin/ |
| 13. | amikacin.mp. or exp Amikacin/ |
| 14. | kanamycin.mp. or exp Kanamycin/ |
| 15. | capreomycin.mp. or exp Capreomycin/ |
| 16. | viomycin.mp. or exp Viomycin/ |
| 17. | enviomycin.mp. or exp Enviomycin/ |
| 18. | fluoroquinolones.mp. or exp Fluoroquinolones/ |
| 19. | levofloxacin.mp. or exp Ofloxacin/ |
| 20. | moxifloxacin.mp. |
| 21. | ofloxacin.mp. |
| 22. | ciprofloxacin.mp. or exp Ciprofloxacin/ |
| 23. | sparfloxacin.mp. |
| 24. | thioamides.mp. or exp Thioamides/ |
| 25. | cycloserine.mp. or exp Cycloserine/ |
| 26. | opc-67683.mp. |
| 27. | PA-824.mp. |
| 28. | thiacetazone.mp. or exp Thioacetazone/ |
| 29. | p-aminosalicylic acid.mp. or exp Aminosalicylic Acid/ |
| 30. | para-aminosalicyclic acid.mp. |
| 31. | ethionamide.mp. or exp Ethionamide/ |
| 32. | rifabutin.mp. or exp Rifabutin/ |
| 33. | rifapentine.mp. |
| 34. | r207910.mp. |
| 35. | terizidone.mp. |
| 36. | protionamide.mp. or exp Prothionamide/ |
| 37. | cilastatin.mp. or exp Cilastatin/ |
| 38. | TMC207.mp. |
| 39. | gatifloxacin.mp. |
| 40. | prothionamide.mp. |
| 41. | or/9-40 |
| 42. | ("health care worker" or "health care workers").ti,ab. |
| 43. | healthy.ti,ab. |
| 44. | 8 or 42 or 43 |
| 45. | ae.fs. |
| 46. | de.fs. |
| 47. | co.fs. |
| 48. | to.fs. |
| 49. | safe.ti,ab. |
| 50. | safety.ti,ab. |
| 51. | side-effect*.ti,ab. |
| 52. | undesirable effect*.ti,ab. |
| 53. | treatment emergent.ti,ab. |
| 54. | tolerability.ti,ab. |
| 55. | toxicity.ti,ab. |
| 56. | adrs.ti,ab. |
| 57. | or/45-56 |
| 58. | 41 and 44 and 57 |
| 59. | exp Case Reports/ |
| 60. | animal/ not human/ |
| 61. | review.pt. |
| 62. | 59 or 60 or 61 |
| 63. | 58 not 62 |

A. Embase

Platform: OvidSP

Database: Ovid Embase 1980-present

Date: 23-08-2011

Limits: no limits were used

Methodological filters: Adverse effect filter: Golder, S. et al Developing efficient search strategies to identify reports of adverse effects in Medline and Embase. Health information and libraries journal (2006),23, p3-12

| **Line #** | **Terms** |
| --- | --- |
| 1. | exp ETHAMBUTOL/ or ethambutol.mp. |
| 2. | pyrazinamide.mp. or exp PYRAZINAMIDE/ |
| 3. | streptomycin.mp. or exp STREPTOMYCIN/ |
| 4. | amikacin.mp. or exp AMIKACIN/ |
| 5. | kanamycin.mp. or exp KANAMYCIN/ |
| 6. | capreomycin.mp. or exp CAPREOMYCIN/ |
| 7. | viomycin.mp. or exp VIOMYCIN/ |
| 8. | enviomycin.mp. or exp ENVIOMYCIN/ |
| 9. | fluoroquinolones.mp. or exp quinolone derivative/ |
| 10. | levofloxacin.mp. or exp LEVOFLOXACIN/ |
| 11. | moxifloxacin.mp. or exp MOXIFLOXACIN/ |
| 12. | ofloxacin.mp. or exp OFLOXACIN/ |
| 13. | exp CIPROFLOXACIN/ or ciprofloxacin.mp. |
| 14. | sparfloxacin.mp. or exp SPARFLOXACIN/ |
| 15. | thioamides.mp. or exp thioamide/ |
| 16. | cycloserine.mp. or exp CYCLOSERINE/ |
| 17. | opc-67683.mp. or exp "2,3 dihydro 2 methyl 6 nitro 2 [4 [4 (4 trifluoromethoxyphenoxy) 1 piperidinyl]phenoxymethyl]imidazo[2,1 b]oxazole"/ |
| 18. | PA-824.mp. or exp "6,7 dihydro 2 nitro 6 (4 trifluoromethoxybenzyloxy) 5h imidazo[2,1 b][1,3]oxazine"/ |
| 19. | thiacetazone.mp. or exp thioacetazone/ |
| 20. | p-aminosalicylic acid.mp. or exp aminosalicylic acid/ |
| 21. | para-aminosalicyclic acid.mp. |
| 22. | ethionamide.mp. or exp ETHIONAMIDE/ |
| 23. | rifabutin.mp. or exp RIFABUTIN/ |
| 24. | rifapentine.mp. or exp RIFAPENTINE/ |
| 25. | r207910.mp. or exp "1 (6 bromo 2 methoxy 3 quinolinyl) 4 dimethylamino 2 (1 naphthyl) 1 phenyl 2 butanol"/ |
| 26. | terizidone.mp. or exp TERIZIDONE/ |
| 27. | protionamide.mp. or exp PROTIONAMIDE/ |
| 28. | exp CILASTATIN/ or cilastatin.mp. |
| 29. | TMC207.mp. or exp "1 (6 bromo 2 methoxy 3 quinolinyl) 4 dimethylamino 2 (1 naphthyl) 1 phenyl 2 butanol"/ |
| 30. | gatifloxacin.mp. or exp GATIFLOXACIN/ |
| 31. | prothionamide.mp. or exp protionamide/ |
| 32. | exp contact examination/ |
| 33. | exp antibiotic prophylaxis/ |
| 34. | exp chemoprophylaxis/ |
| 35. | latent.ti,ab. |
| 36. | contact*.ti,ab. |
| 37. | prevent*.ti,ab. |
| 38. | prophyla*.ti,ab. |
| 39. | ("health care worker" or "health care workers" or ("healthy individual" or "healthy individuals")).ti,ab. |
| 40. | or/32-39 |
| 41. | ae.fs. |
| 42. | to.fs. |
| 43. | co.fs. |
| 44. | safe.ti,ab. |
| 45. | safety.ti,ab. |
| 46. | side-effect*.ti,ab. |
| 47. | undesirable effect*.ti,ab. |
| 48. | treatment emergent.ti,ab. |
| 49. | tolerability.ti,ab. |
| 50. | toxicity.ti,ab. |
| 51. | adrs.ti,ab. |
| 52. | or/41-51 |
| 53. | or/1-31 |
| 54. | exp case study/ |
| 55. | animals/ not humans/ |
| 56. | review.pt. |
| 57. | healthy.ti,ab. |
| 58. | case report/ |
| 59. | 40 or 57 |
| 60. | 53 and 59 |
| 61. | 60 and 52 |
| 62. | 54 or 55 or 56 or 58 |
| 63. | 61 not 62 |
