# Supplementary material for: Adverse Events in Healthy Individuals and MDR-TB Contacts Treated with Anti-Tuberculosis Drugs Potentially Effective for Preventing Development of MDR-TB: A Systematic Review
Source: PLoS One. 2013 Jan 11;8(1):e53599. doi: 10.1371/journal.pone.0053599 (PMC3543458; doi:10.1371/journal.pone.0053599)
Supplement: Supporting Information S2 — Risk of bias assessment. (DOC) [file pone.0053599.s002.doc]

**Annex 2** Summary of risk of bias

**Table 1 Risk of bias of the included randomized controlled trials assessed using the Cochrane Risk of Bias tool**

| **Reference** | **random sequence generation** | **allocation concealment** | **blinding of participants** | **blinding of personnel** | **blinding of outcome assessment** | **incomplete outcome data** | **selective reporting** | **conflict of interest** |
| --- | --- | --- | --- | --- | --- | --- | --- | --- |
| Kraft, 2004 *placebo controlled* *study arm* (61) | **😐** | **😐** | **☺** | **☺** | **😐** | **☺** | **☹** | **☹** |
| Kraft, 2004 *active controlled study arm* (61) | **😐** | **😐** | **☹** | **☹** | **😐** | **☺** | **☹** | **☹** |
| Marier, 2006 (58) | **☺** | **😐** | **☹** | **☹** | **☹** | **☺** | **☹** | **☹** |
| Peeters, 2008 (55) | **☺** | **😐** | **☺** | **☺** | **☺** | **☺** | **☺** | **☹** |
| Stein, 1991 (59) | **😐** | **😐** | **☺** | **☺** | **☺** | **☺** | **☺** | **☹** |
| Sullivan, 1999 (56) | **😐** | **😐** | **☺** | **☺** | **☺** | **☺** | **☺** | **☹** |
| Tsikouris, 2006 (52) | **😐** | **😐** | **☹** | **☹** | **☹** | **☺** | **☹** | **☺** |
| Amsden, 1999 (48) | **😐** | **😐** | **☺** | **☹** | **☹** | **☺** | **☺** | **☺** |
| Ayalasomayajula, 2009 (53) | **😐** | **😐** | **☹** | **☹** | **☹** | **☺** | **☺** | **☹** |
| Burkhardt, 2002 (54) | **😐** | **😐** | **☺** | **☺** | **☺** | **☺** | **☺** | **☹** |
| Chien, 1998 (49) | **😐** | **😐** | **☺** | **☺** | **☺** | **☺** | **☺** | **☹** |
| Chien, 1997 (50) | **😐** | **😐** | **☺** | **☺** | **☺** | **☺^*^** | **☺** | **☹** |
| Chow, 2001 (51) | **😐** | **😐** | **☺** | **☺** | **☺** | **☺** | **☺** | **☹** |
| Ford, 2008 (60) | **😐** | **😐** | **☹** | **☹** | **☹** | **☺** | **☺** | **☹** |
| Guay, 1992(57) | **😐** | **😐** | **☺** | **☺** | **☺** | **☺** | **☺** | **☹** |

Explanation of the symbols used: **☺**: low risk of bias; **😐**: unclear risk of bias; **☹**: high risk of bias.

^*^Although these were done according to the methods section of this paper, results of ophthalmology measurements and electroencephalograms were not given.

Table 2 presents a summary of the risk of bias assessment for the observational studies. The items on comparability were not applicable as all observational studies were non-comparative studies. A study can be awarded a maximum of three stars for Selection and three stars for Outcome. The selection items refer to representativeness of the exposed (treatment) population, measurement of exposure and demonstration that the outcome of interest (adverse effects) was not present at the start of the study. Outcome refers to measurement of the outcome, duration of follow up and completeness of follow up.

**Table 2 Risk of bias of the included observational studies using the Newcastle Ottawa Scale**

| **Reference** | **Selection** | **Outcome** |
| --- | --- | --- |
| *Healthy volunteers* | | |
| Van Saene, 1988 | **🟑** | **🟑** |
| Zhang, 2002 | **🟑🟑** |  |
| *Contacts of MDR-TB patients* | | |
| Horn, 1994 | **🟑🟑🟑** | **🟑🟑** |
| Papastavros, 2002 | **🟑🟑🟑** | **🟑🟑🟑** |
| Ridzon, 1997 | **🟑🟑🟑** | **🟑🟑🟑** |
| Younossian, 2005 | **🟑🟑🟑** | **🟑🟑🟑** |
