# Supplementary material for: Adverse Events in Healthy Individuals and MDR-TB Contacts Treated with Anti-Tuberculosis Drugs Potentially Effective for Preventing Development of MDR-TB: A Systematic Review
Source: PLoS One. 2013 Jan 11;8(1):e53599. doi: 10.1371/journal.pone.0053599 (PMC3543458; doi:10.1371/journal.pone.0053599)
Supplement: Supporting Information S3 — GRADE profiles. (DOCX) [file pone.0053599.s003.docx]

GRADE profiles

| **Quality assessment LEVOFLOXACIN** | | | | | | | **Quality** |
| --- | --- | --- | --- | --- | --- | --- | --- |
|  |  |  |  |  |  |  |  |
| **No of studies** | **Design** | **Risk of bias** | **Inconsistency** | **Indirectness** | **Imprecision** | **Other considerations** |  |
| 6 | randomised trials^1^ | serious^2^ | no serious inconsistency | very serious^3^ | serious^4^ | none | VERY LOW |
|  |  |  |  |  |  |  |  |

^1^ One single arm study; 3 placebo controlled studies; ^2^ Two studies with lack of blinding, 1 study with unclear outcome assessment and all studies had very short study duration.
^3^ Population: healthy volunteers in stead of contacts of MDR-TB patients; Intervention: IV treatment in 3 studies (preventive treatment is oral treatment) and different dosages.
^4^ Small sample size, low number of events

| **Quality assessment MOXIFLOXACIN** | | | | | | | **Quality** |
| --- | --- | --- | --- | --- | --- | --- | --- |
|  |  |  |  |  |  |  |  |
| **No of studies** | **Design** | **Risk of bias** | **Inconsistency** | **Indirectness** | **Imprecision** | **Other considerations** |  |
| 5 | randomised trials | serious^1^ | no serious inconsistency | serious^2^ | serious^3^ | none | VERY LOW |
|  |  |  |  |  |  |  |  |

^1^ Two studies with lack of blinding and all studies had very short study duration; ^2^ Population: healthy volunteers instead of contacts of MDR-TB patients.
^3^ Small sample size, low number of events.

| **Quality assessment OFLOXACIN** | | | | | | | **Quality** |
| --- | --- | --- | --- | --- | --- | --- | --- |
|  |  |  |  |  |  |  |  |
| **No of studies** | **Design** | **Risk of bias** | **Inconsistency** | **Indirectness** | **Imprecision** | **Other considerations** |  |
| 4 | randomised trials^1^ | serious^2^ | no serious inconsistency | very serious^3^ | serious^4^ | none | VERY LOW |
|  |  |  |  |  |  |  |  |

^1^ One single arm study; 2 placebo controlled studies; ^2^ One study with unclear outcome assessment and all studies had very short study duration.
^3^ Population: healthy volunteers instead of contacts of MDR-TB patients; Intervention: IV and oral treatment, different dosages; ^4^ Small sample size, low number of events.

| **Quality assessment RIFABUTIN** | | | | | | | **Quality** |
| --- | --- | --- | --- | --- | --- | --- | --- |
|  |  |  |  |  |  |  |  |
| **No of studies** | **Design** | **Risk of bias** | **Inconsistency** | **Indirectness** | **Imprecision** | **Other considerations** |  |
| 2 | randomised trials | very serious^1^ | no serious inconsistency | serious^2^ | serious^3^ | none | VERY LOW |
|  |  |  |  |  |  |  |  |

^1^ Both studies with lack of blinding and all studies had very short study duration; no placebo-controlled studies.
^2^ Population: healthy volunteers instead of contacts of MDR-TB patients.
^3^ Small sample size, low number of events.
